# Contribution of *elovl5a* to Docosahexaenoic Acid (DHA) Synthesis at the Transcriptional Regulation Level in Common Carp, *Cyprinus carpio*

**DOI:** 10.3390/ani14040544

**Published:** 2024-02-06

**Authors:** Hanyuan Zhang, Peizhen Li, Youxiu Zhu, Yanliang Jiang, Jianxin Feng, Zixia Zhao, Jian Xu

**Affiliations:** 1 Key Laboratory of Aquatic Genomics, Ministry of Agriculture and Rural Affairs, Beijing Key Laboratory of Fishery Biotechnology, Chinese Academy of Fishery Sciences, Beijing 100141, China; lpz18567785189@163.com (P.L.); zhyyx0508@163.com (Y.Z.); jiangyl@cafs.ac.cn (Y.J.); zhaozx@cafs.ac.cn (Z.Z.); 2 Henan Academy of Fishery Sciences, Zhengzhou 450044, China; fnjaxn@163.com; 3 Fisheries Engineering Institute, Chinese Academy of Fishery Sciences, Beijing 100141, China

**Keywords:** DHA, common carp, *elovl5a*, miR-26a-5p, CRISPR/Cas9

## Abstract

**Simple Summary:**

The expression of the elongase of the very-long-chain fatty acid 5 (*elovl5*) gene was investigated in this study using different molecular validation strategies. It has been found that the *elovl5a* gene is a causal gene responsible for DHA content differences in common carp. miR-26a-5P is a casual miRNA that negatively regulates DHA synthesis by targeting the *elovl5a* gene. Considering its down-regulation of the DHA synthesis pathways in common carp, knocking out, knocking down, or silencing the expression of *elovl5a* may increase DHA content in common carp. Overall, our study preliminarily explored the potential function of *elovl5a* in common carp. These results provide useful information for future functional research of the *elovl5* gene and the interactions between its other gene family members in freshwater fish.

**Abstract:**

Docosahexaenoic acid (DHA) is an essential nutrient for humans and plays a critical role in human development and health. Freshwater fish, such as the common carp (*Cyprinus carpio*), have a certain degree of DHA biosynthesis ability and could be a supplemental source of human DHA needs. The elongase of very-long-chain fatty acid 5 (Elovl5) is an important enzyme affecting polyunsaturated fatty acid (PUFA) biosynthesis. However, the function and regulatory mechanism of the *elovl5* gene related to DHA synthesis in freshwater fish is not clear yet. Previous studies have found that there are two copies of the *elovl5* gene, *elovl5a* and *elovl5b,* which have different functions. Our research group found significant DHA content differences among individuals in Yellow River carp (*Cyprinus carpio* var.), and four candidate genes were found to be related to DHA synthesis through screening. In this study, the expression level of *elovl5a* is decreased in the high-DHA group compared to the low-DHA group, which indicated the down-regulation of *elovl5a* in the DHA synthesis pathways of Yellow River carp. In addition, using a dual-luciferase reporter gene assay, we found that by targeting the 3’UTR region of *elovl5a*, miR-26a-5p could regulate DHA synthesis in common carp. After CRISPR/Cas9 disruption of *elovl5a*, the DHA content in the disrupted group was significantly higher than in the wildtype group; meanwhile, the expression level of *elovl5a* in the disrupted group was significantly reduced compared with the wildtype group. These results suggest that *elovl5a* may be down-regulating DHA synthesis in Yellow River carp. This study could provide useful information for future research on the genes and pathways that affect DHA synthesis.

## 1. Introduction

Polyunsaturated fatty acids (PUFAs), which include two categories, omega-3 and omega-6, are essential nutrients for humans. Omega-3 fatty acids play a vital role in human development as phospholipid components that make up the cell membrane structure [1]. As the most important unsaturated fatty acid and a member of omega-3, docosahexaenoic acid (DHA, C22:6n-3) is crucial for promoting brain, retinal, and neural development, especially in babies [2,3]. The development of DHA-related products has great economic prospects.

Deep-sea fish oil is the main source of omega-3 for humans. However, with the annual decline in global marine fishing catch, relying solely on deep-sea fish oil is insufficient to meet the expanding demand for fish oil nowadays. Unlike marine fish, which mainly accumulate PUFA through the food chain, freshwater fish have a certain degree of PUFA biosynthesis ability [4]. Research has found that freshwater fish, such as zebrafish (*Danio rerio*), grass carp (*Ctenopharyngodon idella*), and common carp (*Cyprinus carpio*), have the ability to convert linoleic acid (LA, C18:2n-6) and α-linolenic acid (ALA, C18:3n-3) into DHA [5]. Common carp is one of the most important freshwater aquaculture fish in China. It has a long breeding history, diverse strains, and a wide distribution [6]. Studying the regulatory mechanism of DHA synthesis in common carp is of great significance for developing freshwater fish as a source of DHA.

The biosynthesis of PUFAs in fish is a process in which LA and ALA are used as substrates to synthesize PUFA through continuous desaturation and elongation reactions. This synthesis pathway is mainly catalyzed by fatty acid desaturase (Fads) that catalyzes the desaturation reaction and elongase of very-long-chain fatty acids (Elovl) that catalyze the carbon chain extension reaction [7,8]. The *elovl* gene family, which encodes the synthesis of C18 or longer chain fatty acids [9], is a rate-limiting enzyme in these reaction steps [10], playing a crucial role in fatty acid synthesis [11]. The gene expression level and enzyme activity directly affect the synthesis efficiency of DHA [12].

Analysis of *elovl5* gene sequences in various fish species, including zebrafish [13] and tilapia (*Oreochromis mossambicus*) [14], has found that their amino acid sequences are highly conserved and similar to mammalian characteristic domains. Cloning and expression studies of the *elovl5* gene in freshwater fish, such as zebrafish [14] and rainbow trout (*Oncorhynchus mykiss*) [15], have shown that the *elovl5* gene has different activities in different fish species. Studies of marine fish, such as large yellow croaker (*Larimichthys crocea*) [16], golden pompano (*Trachinotus ovatus*) [17], and Atlantic cod (*Gadus morhua*) [18], have shown that *elovl5* genes in marine fish could extend the substrate of C18 and C20 fatty acids, but lack the ability to convert C22 fatty acids. Overexpression of miR-146a in the liver cell line of rabbitfish (*Siganus canaliculatus*) could inhibit the expression of the *elovl5* gene and thus reduce PUFA content [19]. So far, the function and regulatory mechanism of the *elovl5* gene involved in DHA synthesis in common carp is not clear yet. The molecular regulatory mechanism and specific function of *elovl5* relating to DHA synthesis in common carp need to be further studied.

A previous study has found that there are two highly similar copies of the *elovl5* gene in common carp: *elovl5a* and *elovl5b* [20]. However, there are differences in regulatory mechanisms and functions between these two highly homologous gene copies. They have shown different expression patterns at different development stages, in different tissues, or under different feeding conditions [20]. Our previous research found that there were significant differences in DHA content between individuals from the same population under the same breeding conditions in Yellow River carp (*Cyprinus carpio* var.) [21]. Through genome-wide association analysis (GWAS) and transcriptome differential expression analysis based on DHA content differences in Yellow River carp, combined with analysis of the literature, four genes, including *elovl5a*, acyl-CoA synthetase bubblegum family member 2 (*acsbg2*), fatty acid desaturase 6 (*fads6*), and lipoprotein lipase (*lpl*), were selected as candidate genes potentially related to DHA content [21]. In the present study, we first used real-time fluorescent quantitative PCR (qRT-PCR) to validate the candidate genes screened in the previous study that may be involved inDHA synthesis. After verification, the potential function of *elovl5a* was further explored by dual-luciferase reporter gene assay, CRISPR/Cas9 disruption, and gene expression analysis. The results of this study could improve our understanding of the genetic mechanism of *elovl5a* related to DHA synthesis in common carp and also provide a reference for further research on the interactions between *elovl* gene family members, such as *elovl2* and *elovl5*.

## 2. Methods

### 2.1. Ethics Statement

In this study, all experimental fish were reared and handled following the guidelines for the Care and Use of Experimental Animals of China. Our study was approved by the Animal Care and Use Committee of the Centre for Applied Aquatic Genomics at the Chinese Academy of Fishery Sciences.

### 2.2. Fish Sample and Fatty Acid Content Determination

The Yellow River carp samples (*n* = 96) were collected from the Breeding Station of Henan Academy of Fishery Sciences, Zhengzhou, China. The cultured fish were approximately two years old and had been reared under the same conditions and fed with the same commercial diet (Liaoyang Yida Feed Co., Ltd., Liaoyang, China) before experiment. All the fishes were euthanized in MS222 solution before sampling. According to the experimental design, the muscle and liver tissues of each individual were collected and placed in RNALater (Qiagen, Hilden, Germany), which was kept at 4 °C for 3~6 h to immerse the reagent into the tissue samples, and then immediately frozen in dry ice. Then, 20 g of muscle tissue from each Yellow River carp was collected and stored at −80 °C, until sent to Qingdao Kechuang Testing Co., Ltd. (Qingdao, China) for determination of 35 types of fatty acids content. According to GB/T 22223-2008 standard [22], the samples were processed by gas chromatography with analytical instrument (Agilent, Santa Clara, CA, USA).

### 2.3. RNA Isolation and qRT-PCR

The expression level of DHA content-related genes in Yellow River carp were detected by qRT-PCR. RNA from liver tissue of six fish, each with the highest and the lowest DHA content, were extracted, respectively. cDNAs were synthesized using reverse transcription kit (Qiagen, Shanghai, China). The verified *β-actin* [20] was selected as the internal reference gene. Primer Premier 5 software was used to design qRT-PCR primers based on gene sequences from the common carp genome database (*Cyprinus carpio* Assembly: GCF_000951615.2). The primer sequences are shown in Table 1. Total RNA was reverse transcribed into cDNA using the RevertAid First Strand cDNA Synthesis Kit (Thermo Scientific, Wilmington, DE, USA). The total quantitative experiment system was 15 μL, including 1 μL of cDNA, 0.3 μL per each upper and lower primer, 7.5 μL of SYBR Green Master Mix (TOYOBO, Osaka, Japan), and 5.9 μL of ddH_2_O. The reaction procedure was set at 95 °C for 2 min, followed by 40 cycles at 95 °C for 15 s, 59 °C for 30 s, and 72 °C for 1 min, with the melting curve as the default condition. Each treatment included six biological replicates with three technical duplicates. The relative expression level of each gene was measured by ABI7500 Real-time PCR instrument (Applied Biosystems, Waltham, MA, USA).

### 2.4. Plasmid Construction

To avoid systematic error caused by the negative regulation of mRNA expression in the 3’-untranslated region (3’UTR) of the *elovl5a* gene, in this experiment, using wildtype (WT) as the control group when co-transfecting WT with mimics or inhibitors could better illustrate the regulatory effect of overexpression on the *elovl5a* gene. The Targetscan (http://www.targetscan.org/vert_71/, accessed on 8 April 2022) and AnimalTFDB 3.0 (http://bioinfo.life.hust.edu.cn/AnimalTFDB#!/, accessed on 9 April 2022) were used to predict the putative binding sites of *elovl5a* on the 3’UTR. After obtaining the putative binding sites, the vector was constructed based on the sequences of the *elovl5a* gene 3’UTR region, including putative binding sites. The target region was inserted into the NheI/XhoI site of pmirGLO vector (Genecreate, Nanjing, China) to construct the reporter plasmids (pmirGLO-ELOVL5). The plasmids for transfection were conducted using the TOP10 competent cell (Invitrogen, Waltham, MA, USA) according to the manufacturer’s instructions.

### 2.5. Cell Culture, Transfection, and Dual-Luciferase Report Assay

The vectors were transfected into 293T cells with mimics and mimics NC, respectively. The HEK293T cells were inoculated in the 24-well culture plate the day before transfection and cultured in DMEM high-glucose medium containing 10% FBS in incubator with 5% CO^2^ at 37 °C. miRNA was dissolved in DEPC water to a concentration of 20 μM. Then, a final concentration of 50 of nM miRNA and 2 μg of plasmid were added into 50 μL of serum-free DMEM medium. The mixture was incubated at room temperature for 5 min. After that, 2 μL of lipo2000 transfection reagent was added into 50 μL of serum-free DMEM medium. The mixture was incubated at room temperature for 15 min. Then, the serum-free DMEM medium was supplemented to 0.5 mL and cultured at 37 °C for 4–6 h. We discarded medium and added 0.5 mL of complete medium. pmirGLirGLO-ELOVL5 or pmirGLO-ELOVL5-mut was co-transfected with mimics or mimics NC into SMMC-7721 cells by Lipofectamine-mediated gene transfer. pmirGLO, pmirGLO-ELOVL5, or pmirGLO-ELOVL5-mut was transfected into different SMMC-7721 cell clones and HCCLM6 cell clones via lipofectamine-mediated gene transfer. After 48 h of transfection, the relative luciferase was determined using a dual-luciferase reporter assay system (Genecreate, Nanjing, China) according to the manufacturer’s instructions and normalized to Renilla luciferase activity.

### 2.6. CRISPR/Cas9 Disruption

In order to verify the function of *elovl5a* related to DHA synthesis in Yellow River carp, CRISPR/Cas9 disruption was performed. The ZiFiT website (http://zifit.partners.org/ZiFiT/CSquare9Nuclease.aspx, accessed on 20 April 2022) was used for the sgRNA target design. Sequences of the sgRNA target used for *elovl5a* in this study were 5′-TGCCAGAACACCCACAGTGG-3′. The sgRNA and Cas9 nuclease were synthesized or supplied by Genscript, Nanjing, China. The embryos were randomly divided into inject group and non-inject control group. The injection was prepared using the EasyEdit sgRNA kit from Genscript, Nanjing, China. Immediately after artificial insemination, the fertilized eggs in the single-cell development stage were microinjected using 1 μL injection for inject group. The parameters of the microinjection instrument were adjusted to pi/hpa 467, ti/s 0.1, pc/hpa 18.

### 2.7. Verification after Gene Disruption

The juvenile fish were reared and sampled until seven months old. The fish fillet was collected and stored at −80 °C for DHA content determination and DNA sequencing. The liver tissue was collected and stored in RNA later at −80 °C for qRT-PCR. Marine animal tissue DNA extraction kit (Tiangen, Beijing, China) was used to extract DNA. Primer Premier 5 software was used to design PCR primers for the target region of *elovl5a* gene. The primers are shown in Table 1. Then, the sgRNA target region of *elovl5a* gene was amplified and sequenced. The n-3 PUFA contents of the mutant and wildtype Yellow River carp were detected by Qingdao Kechuang Testing Co., Ltd., Qingdao, China, using gas chromatography–mass spectrometry (GC-MS). The n-3 PUFA typically includes DHA, eicosapentaenoic acid (EPA, C20:5n-3), arachidonic acid (ARA, C20:4n-6), and ALA.

### 2.8. Gene Expression Analysis

RNA was extracted from liver tissue of the mutant and wildtype Yellow River carp. Purity and integrity of total RNA were detected using 1% agarose gel electrophoresis, and cDNA was synthesized using the reverse transcription kit (TOYOBO, Osaka, Japan). Primers for *elovl5a* gene in Table 1 were used for qRT-PCR, and the internal reference gene was *β-actin* [23]. The reaction system, procedure, and statistical analysis are the same as Method 2.2.

### 2.9. Data Analysis

Differences in the relative expression level of each gene between two groups were calculated using the 2^−ΔΔCT^ method with *p*-value < 0.05 (Wilcoxon test) as the threshold. The n-3 PUFA content differences between the mutant group and wildtype group were calculated using *p*-value < 0.05 (*t*-test) as the significant threshold.

## 3. Results

### 3.1. Fatty Acid Content of Yellow River Carp in Muscle Tissue

A total of 96 two-year-old Yellow River carp were sampled and detected for 35 types of fatty acids. Of all types of fatty acids, we are particularly interested in the contents of n-3 PUFA, especially DHA, in this study. The contents of ALA, ARA, EPA, and DHA in the muscle of Yellow River carp ranged from 308.63 to 1971.64 mg/kg, 147.72 to 1063.22 mg/kg, 115.25 to 570.29 mg/kg, and 746.57 to 2257.59 mg/kg, respectively. There were significant differences in the content of four different n-3 PUFA types. DHA has the highest content (1485.54 ± 305.63 mg/kg, mean ± SD, *n* = 96) compared to ALA (906.14 ± 413.28 mg/kg, mean ± SD, *n* = 96) and ARA (481.75 ± 230.85 mg/kg, mean ± SD, *n* = 96), while EPA has the lowest content (258.30 ± 89.83 mg/kg, mean ± SD, *n* = 96). To verify the candidate genes related to the DHA content, six Yellow River carp, each with the highest and lowest DHA content, were selected to conduct the following experiments.

### 3.2. The Expression of Candidate Genes in Yellow River Carp

**Figure 1:**
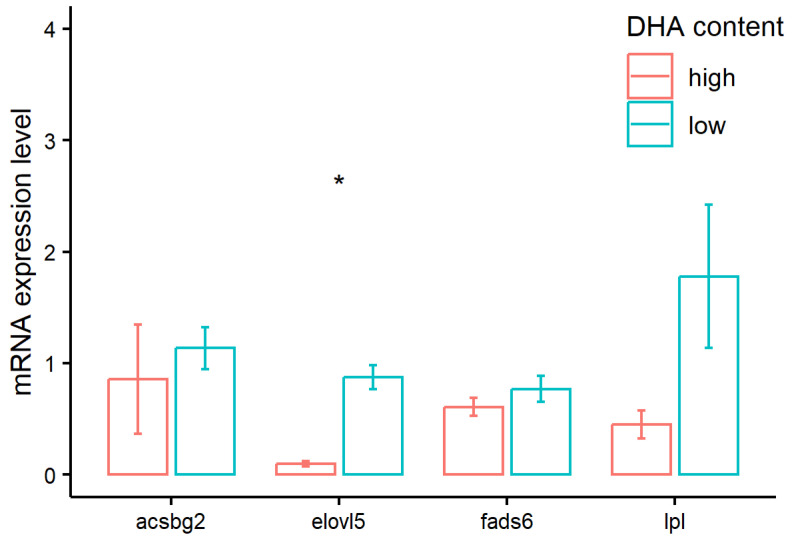
Quantitative real-time PCR based on the relative expression levels of four candidate genes. Data are shown as mean ± SE (*n* = 6). The significant differences were indicated by asterisks (* *p* < 0.05, Wilcoxon test).

As shown in Figure 1, there was no significant differential expression observed in the genes *acsbg2*, *fads6,* and *lpl* between the two groups in the Yellow River carp. Only the expression level of *elovl5a* in the higher DHA content group was significantly lower than those in the lower DHA content group (*p* < 0.05, Wilcoxon test), with the fold-change of −1.99. Even though the difference in expression level between the two groups in the *lpl* gene seemed large, the statistical result was non-significant. This could be caused by the small sample size and the large sample variance within the group.

### 3.3. elovl5a Is the Target Gene of miR-26a-5p

**Figure 2:**
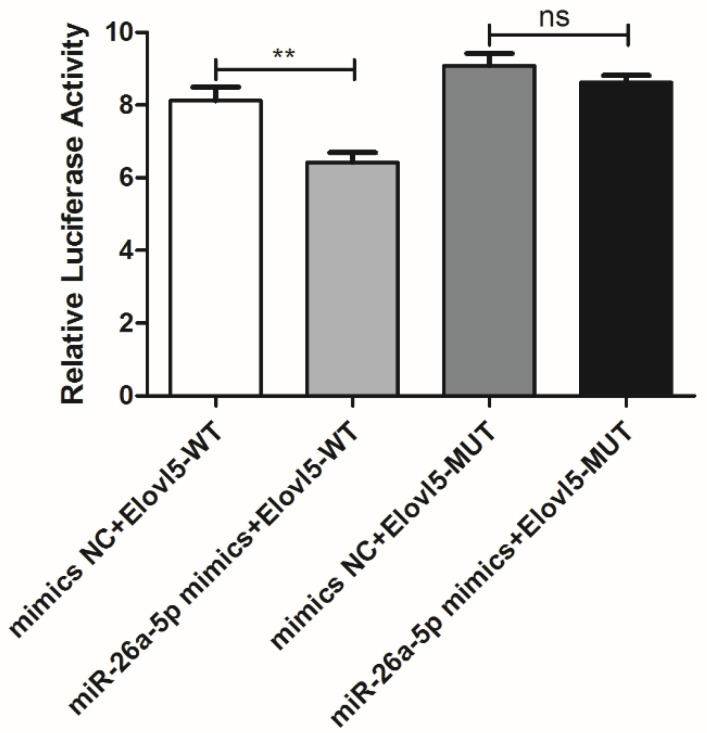
Results of the targeting relationship between miR-26a-5p and *elovl5a*. The firefly/Renilla activity was measured when miR-26a-5p mimics or mimics NC with wildtype (Elovl5-WT) or mutant (Elovl5-MUT) pmir-GLO vectors were co-transfected into HEK 293t cells for 48 h. Data are shown as mean ± SE (*n* = 3). The significant differences were indicated by asterisks (** *p* < 0.01, ns *p* > 0.05, Wilcoxon test).

With regard to putative binding sites predicting miR-26a-5p, the sequence of which was paired with the 3’UTR region of the *elovl5a* gene, they were found to be related to fatty acid synthesis. To elucidate the underlying mechanism of miR-26a-5p in the DHA content regulation in Yellow River carp, the interaction between miR-26a-5p and the 3’UTR of *elovl5a* was investigated. The seed region of miR-26a-5p exhibited a specific complementary binding site to the 3’UTR region of *elovl5a*, including seven nucleotides (miR-26a-5p: 3′-UUGGAUAGGACCUAAUGAACUU-5′, *elovl5a*‒3’UTR: 5′-AAUAUCUUCAUCUUUACUUGAU-3′). Wildtype and mutant dual luciferase reporter vectors of the *elovl5a* gene were constructed: pmir-GLO-ELOVL5 WT 5′-AATATCTTCATCTTTACTTGAT-3′, pmir-GLO-ELOVL5 MUT 5′-AATATCTTCATCTTATGAACTT-3′. The vectors were transfected into 293T cells with miR-26a-5p mimics and mimics NC, respectively. After determination by dual-luciferase reporter assay and normalization, Figure 2 showed that the co-transfection of Elovl5-WT with miR-26a-5p mimics resulted in an extremely significant reduction in the relative luciferase activity (*p* < 0.01, Wilcoxon test), while co-transfection with Elovl5-MUT did not induce a significant change in luciferase activity. These results suggested that *elovl5a* was a target gene of miR-26a-5p, and miR-26a-5p negatively regulated the expression of the *elovl5a* gene.

### 3.4. CRISPR/Cas9 Disruption of elovl5a in Yellow River Carp

**Figure 3:**
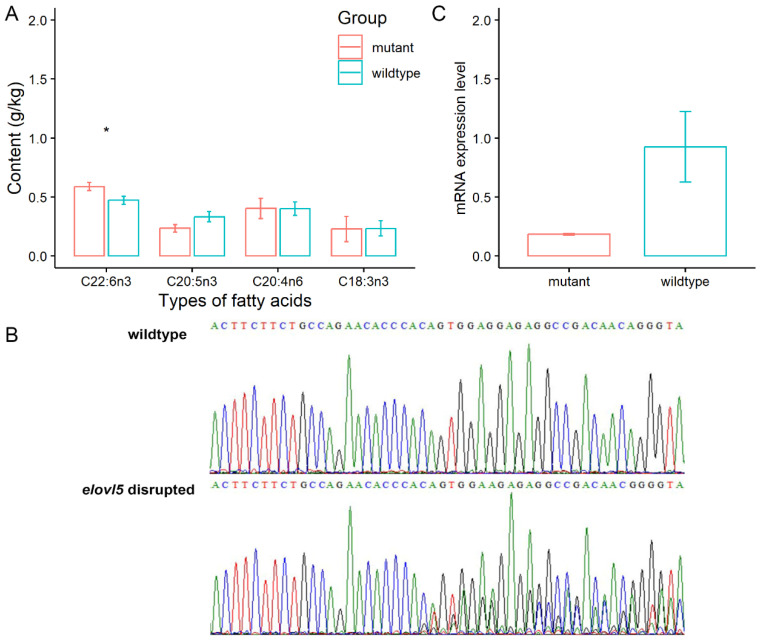
Results of *elovl5a* gene disruption between wildtype and mutant Yellow River carp. (**A**) The n-3 PUFA content differences between wildtype and mutant Yellow River carp. Data are shown as mean ± SE (*n* = 6). The significant differences were indicated by asterisks (* *p* < 0.05, *t*-test). (**B**) The mutations in the *elovl5a*-disrupted individuals were detected by Sanger sequencing. (**C**) The mRNA expression level of wildtype and *elovl5a*-disrupted individuals. Data were shown as mean ± SE (*n* = 3).

To further verify the DHA content-related function of *elovl5a* in Yellow River carp, CRISPR/Cas9 disruption was conducted by microinjection into the fertilized eggs. The n-3 PUFA content of mutant and wildtype Yellow River carp is shown in Table 2. There were no statistical differences between the non-injected group and the injected group in the content of ALA (C18:3n3), ARA (C20:4n6), and EPA (C20:5n3). However, the DHA (C22:6n3) content was significantly higher in the injected group compared with the non-injected group (*p* < 0.05, *t*-test, Figure 3A). In addition, there were no statistically significant differences in body weight and length between the non-injected group and the injected group from the same cage (*p* > 0.05).

After sequencing verification of the sgRNA target region of the *elovl5a* gene, no gene modifications or mutations were observed in the non-injected group, whereas in the injected group, nested peaks within the target region were observed in the mutant individuals, indicating a relatively high frequency of mutations. Two representative sequencing results of PCR products, which corresponded to the wildtype and *elovl5a* gene disrupted individuals, were shown in Figure 3B. The sequencing results of the wildtype (*wt*) individuals were completely consistent with the theoretical *elovl5a* sgRNA sequences (TGCCAGAACACCCACAGTGG). However, hybrid peaks started from the sgRNA target site in the mutant (*mu*) individuals. Using DNAMAN 9.0 software to compare *wt* and *mu* amplified sequences, it was found that mutated individuals undergo frameshift mutations starting from the sgRNA target, indicating a possible change in the protein function. Six individuals each from the *wt* and *mu* groups were selected to conduct qRT-PCR experiments. The relative expression level of the *elovl5a* gene is shown in Figure 3C. Compared with the *wt* group, the expression level of the *elovl5a* gene in the *mu* group was significantly reduced (*p* < 0.01).

## 4. Discussion

PUFAs play an important role in the growth, development, and reproduction of fish and are also of great significance to human health [24,25]. In particular, the content of DHA is an important indicator representing the quality of fish. Freshwater fish have a certain degree of PUFA biosynthesis ability, which is worth our in-depth research. Previous studies have reported that the *fads* and *elovl* gene families are the two main gene families affecting DHA synthesis in teleosts [26,27]. Screening and identifying genes that affect DHA synthesis at the genomic level can significantly shorten the breeding time compared with traditional breeding methods. Therefore, searching for the main genes that affect DHA content would benefit the improvement in fish quality by conducting molecular breeding.

In the present study, qRT-PCR was conducted to identify the DHA content-related genes (including *acsbg2*, *elovl5a*, *fads6*, and *lpl*) in Yellow River carp. A significantly differentially expressed gene, *elovl5a,* was identified, indicating that the *elovl5a* gene may down-regulate the DHA synthesis in Yellow River carp. To verify the potential down-regulation of *elovl5a* in the DHA synthesis processes in Yellow River carp, a dual-luciferase reporter gene assay was performed, and we found that miR-26a-5p may negatively regulate the DHA synthesis by targeting the 3’UTR region of *elovl5a*. Furthermore, the contribution of *elovl5a* to the DHA content in Yellow River carp was verified by the CRISPR/Cas9 gene editing system. The DHA content in the disrupted group was significantly increased, while the expression level of *elovl5a* was significantly reduced in the disrupted group compared with the wildtype group, suggesting that the *elovl5a* gene down-regulates DHA synthesis in Yellow River carp.

Results from qRT-PCR, dual-luciferase reporter gene assay, and gene editing experiments all indicated that *elovl5a* negatively regulates DHA synthesis in Yellow River carp. These results are consistent with recent studies on the function of the *elovl5* gene in zebrafish. Compared with wildtype (*elovl5*^+^) zebrafish, the C22 PUFA content of *elovl5* knockout (*elovl5*^−^) zebrafish was significantly increased, which may be related to the up-regulation of *elovl4b* and *elovl2* expression [28]. Meanwhile, the expression level of *elovl5* in the liver of *elovl2* knockout (*elovl2*^−^) zebrafish is quite low, suggesting that *elovl5* may play a role as an “assistant attacker” of *elovl2* in the LC-PUFA synthesis of zebrafish [28]. Another zebrafish *elovl2*/*elovl5* comparative gene knockout experiment showed that Elovl2, but not Elovl5, is essential for DHA biosynthesis in zebrafish [29]. The liver DHA content of the *elovl2*^−^ mutant was significantly reduced, while the *elovl5*^−^ mutant was not significantly changed, indicating that the endogenous synthesis of DHA in zebrafish is mediated by Elovl2.

It is necessary to further discuss another member of the *elovl* gene family, *elovl2*, whose function is similar to *elovl5*. The *elovl2* gene has been lost in many marine fish and has only been cloned in a few fish species, such as zebrafish, Atlantic salmon (*Salmo salar*), and rainbow trout [11,30]. In zebrafish, heterologous expression of the *elovl2* gene can increase the content of Docosapentaenoic acid (C22:5n-3), the precursor of DHA [31]. In cyprinid fish, the correlation between the expression level of *elovl2* and *elovl5* is significantly higher than that of other members of the *elovl* family. Previous studies have shown that there was a complex interaction between Elovl2 and Elovl5 enzymes. In teleost, *elovl2* and *elovl5*, as collateral homologous genes, have similar substrate activity. The Elovl2 and Elovl5 in zebrafish and grass carp both possess the activity of extending C18 and C20 substrates to synthesize PUFA [14]. There is a potential synergistic or competitive relationship between the two enzymes in catalyzing the biosynthesis pathway of PUFAs [32,33]. Therefore, researchers conducted gene editing experiments on the *elovl2* and *elovl5* genes and found that after the disruption of the *elovl2* gene in zebrafish and Atlantic salmon, DHA synthesis in liver and muscle was inhibited and the content was significantly reduced, while EPA accumulated significantly [28,29,34]. However, there was no significant change in the DHA content of the *elovl5*-disrupted zebrafish, indicating that *elovl2*, rather than *elovl5*, plays a key role in the DHA synthesis of zebrafish. Our research confirmed that *elovl5a* is not the key gene to promote DHA synthesis in Yellow River carp, but may inhibit DHA synthesis. This may be related to the competition between *elovl2* and *elovl5* for substrates, or it may be due to the lack of catalytic activity of *elovl5a* for DHA precursor substances in Yellow River carp. Studies on zebrafish and large yellow croaker showed that the conversion efficiency of Elovl5 to different fatty acid substrates decreased with the increase in substrate carbon chain length (conversion rate C18 > C20 > C22) [14,35]. Yeast heterologous expression experiments indicated that *elovl5* preferred to catalyze the elongation reaction of C18 PUFA, while *elovl2* has a higher efficiency in catalyzing the synthesis of DPA from C20 and C22 PUFA substrates [32,36,37]. In addition, as carp are allotetraploid fish, there are two copies of the *elovl5* gene [20,38,39]. Therefore, further studies on the functions of *elovl5b* and *elovl2* genes, as well as the interactions among *elovl* gene family members, will help us to better understand the functions of the *elovl* gene family in the synthesis of DHA and PUFA in carp.

Overall, our study revealed that the *elovl5a* gene is a causal gene responsible for the DHA content differences in Yellow River carp. miR-26a-5P may be a casual miRNA that negatively regulates DHA synthesis by targeting the *elovl5a* gene. Considering its down-regulation of the DHA synthesis in Yellow River carp, knocking out, knocking down, or silencing the expression of *elovl5a* could increase the DHA content in Yellow River carp.

## 5. Conclusions

Previously, we identified *elovl5a*, *acsbg2*, *fads6*, and *lpl* as the candidate genes which related to the DHA content of common carp. In this study, the expression level of *elovl5a* was significantly higher in the high-DHA content carp compared with the low-DHA content group. Furthermore, we found that a key miRNA, miR-26a-5p, may act as an important factor in the regulation of DHA synthesis in Yellow River carp by targeting the 3’UTR region of *elovl5a*. Moreover, the DHA content in the *elovl5a*-disrupted group is significantly increased compared to the wildtype group. Meanwhile, the expression level of *elovl5a* was significantly down-regulated in the *elovl5a*-disrupted fish compared with the wildtype group. To sum up, our study preliminarily explored the potential function of *elovl5a* and found a new miRNA closely related to the regulation of DHA content in Yellow River carp. These results provide useful information for the future functional research of the *elovl5* gene and the interactions between its other gene family members.

## Figures and Tables

**Table 1:** Primers used in this study.

Gene	Primer Sequences (5′‒3′)	Product Length (bp)	Purpose
*acsbg2*	F: CTCAAGTGTAACGTTGATGACAATG	237	qRT-PCR
	R: AGATATGGAGAAGTCTCGTGGAAG		
*elovl5a*	F: CAGGATGATGAACGTACTTTGGTG	209	qRT-PCR
	R: GGATGAAGCTGTTAAACGTGGC		
*fads6*	F: GTGTGTGTGTCTGGGGTTTTTT	198	qRT-PCR
	R: GTCATTTGGTAGATGCGTTTGG		
*lpl*	F: GAGTCAACAAAATTCGCACACG	205	qRT-PCR
	R: TTCAAAGCAGGCATAATGTAGGG		
*elovl5a*	F: GCTTTGAACATATTTCCTTCCTGAC	879	PCR
	R: TGCCCAATTCACCACTGCTT		

**Table 2:** The statistical description of n-3 PUFA content in mutant and wildtype Yellow River carp.

Trait	Min (g/kg)	Max (g/kg)	Average (g/kg)	SD	Coefficient of Variation (%)
ALA—mutant	0.062	0.751	0.228	0.024	103.9
ALA—wildtype	0.075	0.444	0.233	0.014	61
ARA—mutant	0.228	0.802	0.403	0.019	47.88
ARA—wildtype	0.235	0.58	0.401	0.013	31.63
EPA—mutant	0.135	0.322	0.235	0.016	53.12
EPA—wildtype	0.248	0.449	0.332	0.010	20
DHA—mutant	0.506	0.668	0.589	0.007	12.37
DHA—wildtype	0.343	0.537	0.472	0.007	14.54

## Data Availability

The data presented in this study are available on request from the corresponding author.
